# Pharmacological evaluation of new constituents of “Spice”: synthetic cannabinoids based on indole, indazole, benzimidazole and carbazole scaffolds

**DOI:** 10.1007/s11419-018-0415-z

**Published:** 2018-04-26

**Authors:** Clara T. Schoeder, Cornelius Hess, Burkhard Madea, Jens Meiler, Christa E. Müller

**Affiliations:** 1 PharmaCenter Bonn, Pharmaceutical Institute, Pharmaceutical Chemistry I, University of Bonn, An der Immenburg 4, 53121 Bonn, Germany; 2 Institute of Forensic Medicine, Forensic Toxicology, University Hospital of Bonn, Stiftsplatz 12, 53111 Bonn, Germany; 3 Research Training Group 1873, University of Bonn, 53127 Bonn, Germany; 4 Departments of Chemistry and Pharmacology, Vanderbilt University, Stevenson Center, Station B 351822, Nashville, TN 37235 USA

**Keywords:** Pharmacological evaluation of new synthetic cannabinoids, Affinities for CB_1_ and CB_2_ receptors, β-Arrestin assay at GPR18 and GPR55, cAMP accumulation assay, Benzimidazole and carbazole, Structure-activity relationships

## Abstract

**Purpose:**

In the present study we characterized a series of synthetic cannabinoids containing various heterocyclic scaffolds that had been identified as constituents of “Spice”, a preparation sold on the illicit drug market. All compounds were further investigated as potential ligands of the orphan receptors GPR18 and GPR55 that interact with some cannabinoids.

**Methods:**

The compounds were studied in radioligand binding assays to determine their affinity for human cannabinoid CB_1_ and CB_2_ receptors expressed in CHO cells, and in cAMP accumulation assays to study their functionality.

**Results:**

Structure-activity relationships were analyzed. The most potent CB_1_ receptor agonist of the present series MDMB-FUBINACA (**12**) (*K*_i_ = 98.5 pM) was docked into the human CB_1_ receptor structure, and a plausible binding mode was identified showing high similarity with that of the co-crystallized THC derivatives. MDMB-CHMCZCA (**41**) displayed a unique profile acting as a full agonist at the CB_1_ receptor subtype, but blocking the CB_2_ receptor completely. Only a few weakly potent antagonists of GPR18 and GPR55 were identified, and thus all compounds showed high CB receptor selectivity, mostly interacting with both subtypes, CB_1_ and CB_2_.

**Conclusions:**

These results will be useful to assess the compounds’ toxicological risks and to guide legislation. Further studies on **41** are warranted.

**Electronic supplementary material:**

The online version of this article (10.1007/s11419-018-0415-z) contains supplementary material, which is available to authorized users.

## Introduction

A challenging issue for forensic toxicologists and law makers is how to effectively respond to the constantly changing new psychoactive substances on the illicit drug market [1]. Among these, synthetic cannabinoids feature prominently [2, 3]. Between 2008, when so-called “Spice” products [4] containing synthetic cannabinoids began to appear on the drug market, and 2016, 169 new synthetic cannabinoids were confiscated and identified [2]. Most of them were discovered as powders, often in bulk amounts, while others were found in preparations of plant materials, e.g., minced herbs, onto which solutions of the cannabinoids had been sprayed [5]. These substances have been shown to bind to and in many cases activate cannabinoid (CB) receptors. CB receptors are divided into two subtypes, CB_1_ and CB_2_, which belong to the large family of rhodopsin-like class A G protein-coupled receptors (GPCRs) [6]. Both CB receptor subtypes are coupled to G_i_ proteins including a reduction in intracellular cAMP levels. The main psychoactive effects of cannabinoids are mediated by the CB_1_ receptor, which is predominantly expressed in the central nervous system [7], while CB_2_ receptor expression in the brain is restricted to microglial cells [8, 9]. CB_2_ receptors are highly expressed in the immune system, for example in tonsils and spleen [10, 11]. Activation of the CB_2_ receptor is considered as a new therapeutic option for the treatment of inflammatory diseases and pain [12, 13].

**Fig. 1:**
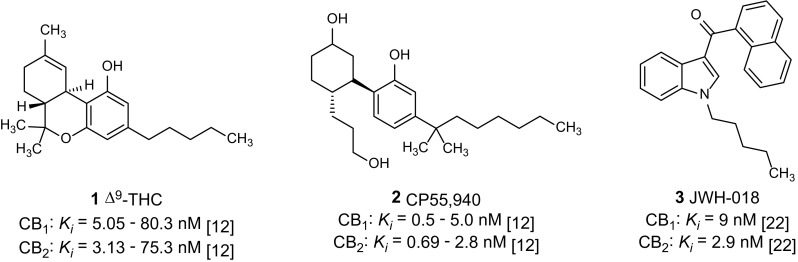
Standard cannabinoid CB_1_/CB_2_ receptor agonists [12, 22]

The plant-derived partial CB_1_ and CB_2_ receptor agonist Δ^9^-tetrahydrocannabinol (Δ^9^-THC, **1**, Fig. 1) is used in therapy to target muscle spasms, nausea and cachexia [14]. The synthetic compound CP55,940 (**2**, Fig. 1) represents a potent full agonist at both receptor subtypes. A CB_1_ receptor antagonist, rimonabant, had been approved for the treatment of obesity but was later withdrawn from the market due to side effects resulting in depression and an increased suicide rate [15].

**Fig. 2:**
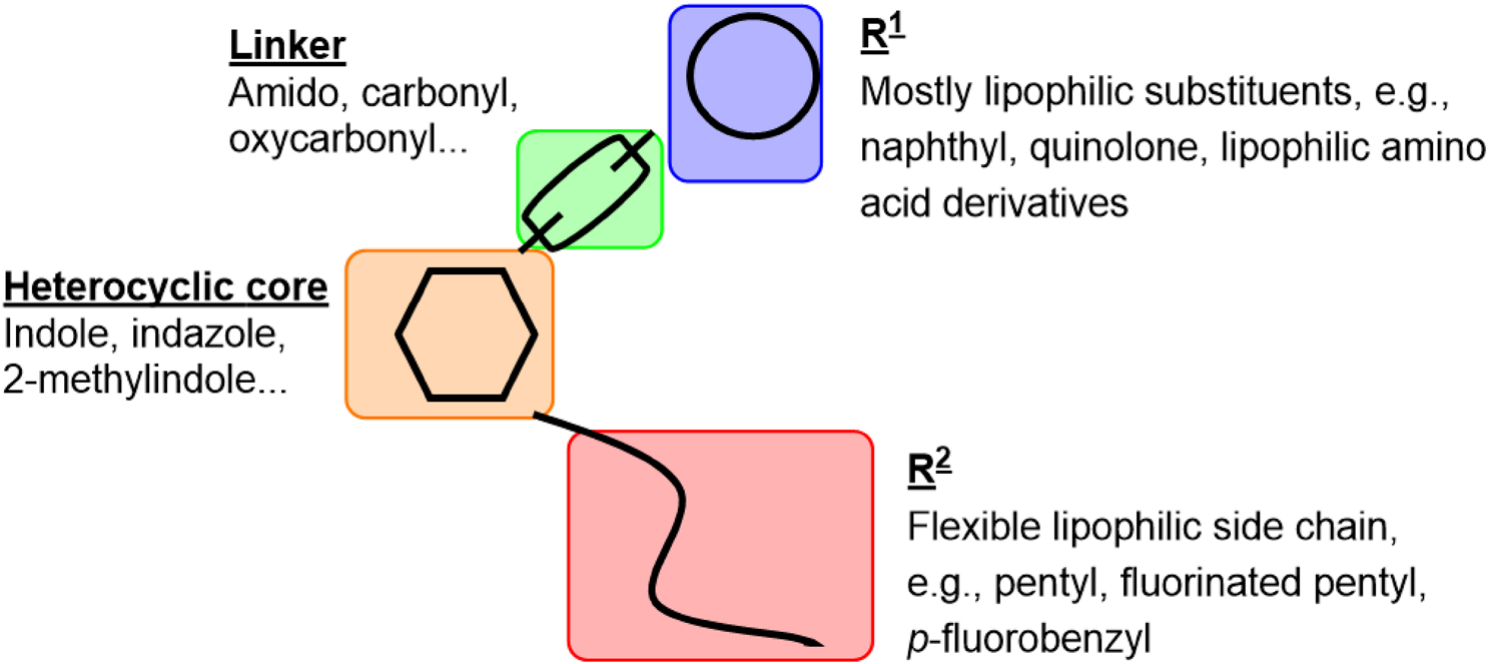
Common structural features of synthetic cannabinoids. The figure was adopted from the European Monitoring Centre for Drugs and Drug Addiction (EMCDDA) [3] and modified

The prevalence for the use of illegal psychoactive substances in Europe by 15–16 year-old teenagers was estimated in 2015 to be about 4% [5]. Synthetic CB_1_ receptor agonists are abused as an alternative to natural marijuana due to their psychoactive and analgesic effects. For synthetic cannabinoids more and more severe side effects and intoxications are reported; they are predominantly neurologic symptoms, but acute organ toxicity has also been observed [16]. In the USA, the principle of enumeration is used to restrict newly discovered synthetic cannabinoids, and every single synthetic cannabinoid has to be individually listed by name in the US List of Schedule I drugs [17]. In Germany new synthetic cannabinoids are legally controlled since November 2016 when the “Neue-Psychoaktive-Stoffe-Gesetz” (NpSG, New Psychoactive Substances Act) was adopted in [18]. Similar regulations exist in Austria and Switzerland [19, 20]. All corresponding compounds, the chemical structures of which are represented by a general formula in the statute with known structure-activity relationships (SARs), were restricted. Newly discovered SARs of synthetic cannabinoids will, therefore, provide a basis for future amendments. However, in many cases, only limited information is available regarding the activity of new substances. Both the affinity of a drug for its receptor and its ability to produce an agonistic response are important features, and these should be determined according to a compound’s chemical structure. For important classes of synthetic cannabinoids, at least four structural components, which have firstly been described by Huffman et al. and were later refined by the European Monitoring Centre for Drugs and Drug Addiction (EMCDDA), are of importance (see Fig. 2 [3]): (1) a heterocyclic core consisting of indole or indazole with different substitutions; (2) a linker, e.g., an ester, amide or ketone; (3) a bulky lipophilic residue (R^1^), e.g., a heterocyclic or aryl substituent, but in newer synthetic cannabinoids a lipophilic amino acid can also be found; and (4) a residue (R^2^) which is a hydrophobic “side chain” attached to the nitrogen atom of the indole or the indazole ring system [21, 22]. The compound JWH-018 (**3**, see Fig. 1), a potent CB_1_ and CB_2_ receptor agonist, displays the basic features of this compound class and was one of the first synthetic cannabinoids identified in herbal blends for abuse [23, 24]. The common features of known synthetic cannabinoids are depicted in Fig. 2.

In a previous study [25], we had determined the pharmacological properties of 48 synthetic cannabinoids collected by the Institute of Forensic Medicine of the University of Bonn. In the present study, we investigated the affinities and functional properties of a new series of 42 synthetic cannabinoids, 16 of which have not been reported as cannabinoid receptor ligands before. The investigated set of compounds comprises four different core structures. The first three groups (A, B, C, see Table 1) represent differently substituted indoles and indazoles, which are structurally derived from the synthetic cannabinoids previously introduced by Huffman et al. and are widely distributed in illicitly sold "Spice" products. In the current study we investigated compounds with l-valinamide (AB)/l-*tert*-leucinamide (ADB or MAB), methyl-3,3-dimethylbutanoate (MDMB), methyl-3-methylbutanoate (MMB), and 2-methyl-2-phenylpropyl (cumyl) moieties as substituents in the R^1^ position. Further classes of compounds consist of carbazoles (E), substituted in position 3, and benzimidazole derivatives (F).

**Table 1:**
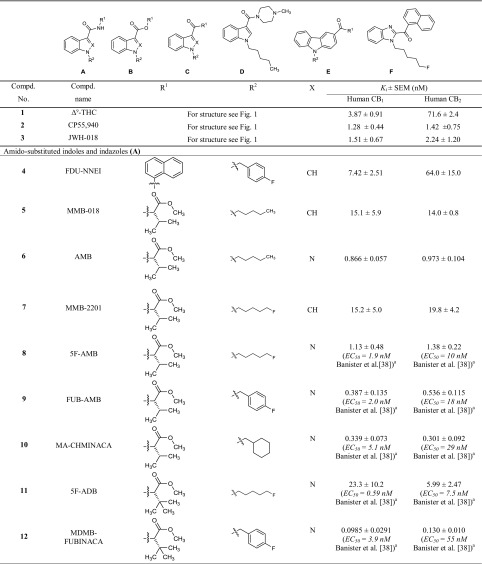

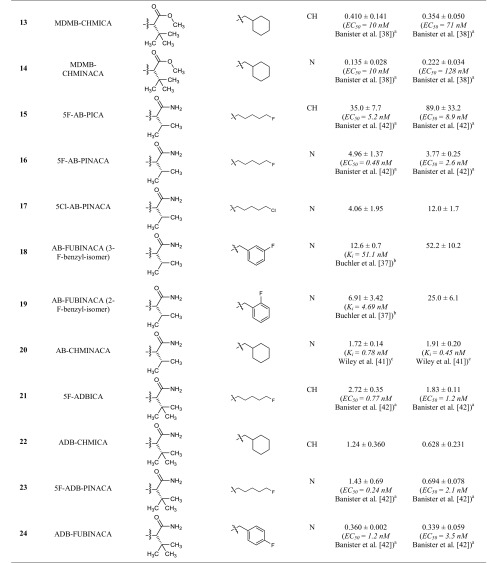

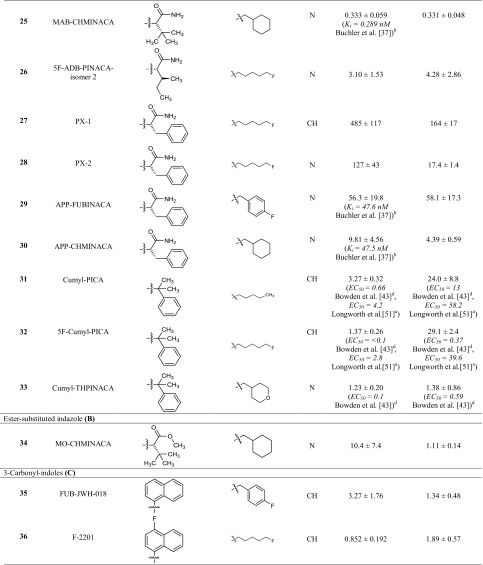

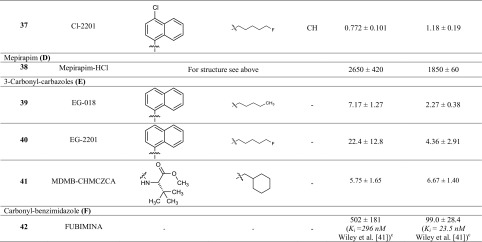
Affinities of the investigated indoles, indazoles and carbazoles at the cannabinoid CB_1_ and CB_2_ receptors determined in radioligand binding assays

^a^Fluorometric imaging plate reader membrane potential assay system from Molecular Devices (Sunnyvale, CA, USA)

^b^Radioligand binding study versus 0.5 nM [^3^H]CP55,940

^c^Radioligand binding study versus 0.62 nM [^3^H]CP55,940

^d^Homogenous time resolved fluorescence-based cAMP accumulation

Radioligand binding and cAMP functional studies on CB_1_ and CB_2_ receptors were complemented by CB_1_ receptor modeling and docking of the most potent CB_1_ receptor agonist of the present series to predict its interactions. We further tested all compounds for their ability to activate or block the two orphan GPCRs GPR18 and GPR55, both of which are known to interact with cannabinoids [26–29]. We discuss SARs of the newly investigated compounds, integrating previously reported data, thereby providing a comprehensive analysis, which will help to predict properties of novel derivatives.

## Methods

### Compounds

All compounds except for MDMB-CHMCZCA (**41**) were obtained from Cayman Chemical (Ann Arbor, MI, USA). According to the manufacturer, the purity of all compounds was declared to be > 95% as determined by liquid chromatography–tandem mass spectrometry (LC–MS/MS). MDMB-CHMCZCA (**41**) was purchased from www.brc-finechemicals.com. We confirmed the purity of all compounds in our laboratories by liquid chromatography–ultraviolet-mass spectrometry (LC–UV-MS) measurements and found it to be generally ≥ 96%, except for MDMB-FUBINACA (**12**, 93%) and Cl-2201 (**37**, 86%).

### Radioligand binding assays

Radioligand binding assays were performed as described previously [25]. Membrane preparations of Chinese hamster ovary (CHO) cells overexpressing the human CB receptor subtype CB_1_ or CB_2_ were incubated in the presence of the test compound and the radioligand [^3^H]CP55,940 (0.1 nM, see Fig. 1) (Perkin-Elmer Life Sciences, Rodgau-Jügesheim, Germany), for 2 h. Bound and unbound radioligand were separated by rapid filtration through glass fiber GF/C-filters (Perkin-Elmer, Boston, MA, USA), using a Brandel 96-well Harvester (Brandel, Gaithersburg, MD, USA). Radioactivity on the filters was determined by liquid scintillation counting. Three separate experiments were performed, each in duplicates.

### cAMP accumulation assays

cAMP accumulation assays were performed also as previously described [25]. Briefly, CHO cells stably expressing the respective human CB receptor subtype CB_1_ or CB_2_ were seeded overnight. Then the phosphodiesterase inhibitor Ro-20-1724 [4-(3-butoxy-4-methoxyphenyl)methyl-2-imidazolidone; Sigma-Aldrich, St. Louis, MO, USA], and subsequently the test compound (10–1 µM) and forskolin (10 µM, Sigma-Aldrich) were added. After incubation for 15 min the buffer was removed, and the cells were lyzed. The amount of cAMP was determined in a radioligand binding assay by incubating 50 µL of the cell lysate with 3 nM [^3^H]cAMP in the presence of protein preparations from bovine adrenal glands (cAMP binding protein) [30]. Bound and unbound radioligand were separated by rapid filtration through GF/B filters, and radioactivity was determined by liquid scintillation counting. To test for antagonistic activity, test compounds were added to Hank’s buffered salt solution (HBSS) containing 10% dimethyl sulfoxide (DMSO), 10 min after the application of Ro-20-1724, and the mixture was incubated for 20 min at 37 °C. Then, the CB agonist CP55,940 was added at a concentration of 0.03 µM, and cAMP determination was carried out as described above [25].

### β-Arrestin assays

β-Arrestin assays were performed in recombinant CHO cells expressing either the human GPR18 or the human GPR55 as described before using the β-galactosidase enzyme fragment complementation technology (β-arrestin PathHunter™ assay; DiscoverX, Fremont, CA, USA) [25].

### Data analysis

Data were analyzed using GraphPad Prism Version 4.02-6.1, (GraphPad Software, San Diego, CA, USA).

### Molecular docking

Molecular docking studies were carried out with the software package Rosetta (www.RosettaCommons.org) using the 2017.08.59291 build [31, 32]. As templates the X-ray structures 5XRA and 5XR8 were employed [33]; fusion proteins and ligands were deleted and a conformer of MDMB-FUBINACA (**12**) was manually positioned in an initial model using the PyMOL Molecular Graphics System, Version 1.7.4.5 (Schrödinger, Inc., New York, NY, USA). A conformer library of MDMB-FUBINACA (**12**) was calculated using the BCL Conformer:Generator [34]. Docking procedure and scripts for data processing are described in supplementary material. Docking scores were calculated using the Rosetta InterfaceAnalyzer. The best scoring models were clustered into a set of plausible binding poses. Results were compared to the pose of THC-like agonists in the template crystal structures 5XRA and 5XR8 and displayed using UCSF Chimera [35].

## Results and discussion

### Cannabinoid CB_1_ and CB_2_ receptor affinities

In the present study, CB_1_ and CB_2_ receptor affinities of a new series of synthetic cannabinoids were determined in radioligand binding studies, which provide an ideal basis for the analysis of SARs (Table 1). The investigated compounds comprise indole, indazole, benzimidazole and carbazole derivatives. For some of the compounds, EC_50_ values had previously been determined by functional assays; however, functional data are highly dependent on the expression level of the receptors or “receptor reserve”, while *K*_i_ values obtained in binding studies are largely independent of the employed cellular background [36].

The present set of compounds includes amino acid derivatives. These types of compounds were originally described in a patent and claimed as potential pain therapeutics [37]. In all cases, an alkyl or heteroaryl residue was introduced as R^2^, and the amino acid was coupled to an amino group in the R^1^ position (see Table 1; Fig. 2) [37]. The presented compounds feature a pentyl or 5-fluoropentyl side chain in position R^2^ (for Table 1; Fig. 2). MMB-018 (**5**), an indole derivative substituted with a valine methyl ester, showed affinity in the low nanomolar range with a *K*_i_ value of 15.1 nM at the CB_1_ receptor and an almost identical *K*_i_ value of 14.0 nM at the CB_2_ receptor. The corresponding indazole AMB (**6**) was more potent displaying subnanomolar affinity for both CB receptor subtypes (CB_1_ *K*_i_ = 0.866 nM; CB_2_ *K*_i_ = 0.973 nM), indicating the superiority of the indazole core. The 5-fluoropentyl derivatives MMB-2201 (**7**) and 5F-AMB (**8**) were similarly as potent as their pentyl analogues MMB-018 (**5**) and AMB (**6**), respectively, showing that the terminal fluorination of the pentyl side chain gives almost no effect. Compounds with a *p*-fluorobenzyl residue or a bioisosteric cyclohexylmethyl residue showed increased affinities in the subnanomolar range in the indazole series (FUB-AMB (**9**), CB_1_ *K*_i_ = 0.387 nM, and MA-CHMINACA (**10**), CB_1_ *K*_i_ = 0.339 nM) and were about equipotent at the CB_2_ receptor. Banister et al. [38] had already investigated these compounds and also 5F-AMB (**8**) in a fluorescence-based membrane potential assay and determined potencies in the nanomolar range (EC_50_ values ranging from 1.9 to 71 nM) in that assay, while our radioligand binding assay revealed higher affinities.

The valine methyl ester was replaced by a *tert*-leucine methyl ester in four of the investigated compounds: 5F-ADB (**11**), MDMB-FUBINACA (**12**), MDMB-CHMICA (**13**) and MDMB-CHMINACA (**14**), substituted with each 5-fluoropentyl (**11**), *p*-fluorobenzyl (**12**) and cyclohexylmethyl residue (**13**,**14**) for R^2^, respectively. MDMB-FUBINACA (**12**) was the most potent compound of the entire set of investigated compounds with a *K*_i_ value of 0.0985 nM at the CB_1_ receptor and 0.130 nM at the CB_2_ receptor. Banister et al. [38] had reported EC_50_ values of 3.9 nM at CB_1_ and of 55 nM at CB_2_ receptors determined in a fluorescence-based membrane potential assay for this compound [38]. MDMB-FUBINACA had caused the highest hypothermal response which the authors had ever observed in rats [38]. These results showed once more that functional assays often do not correctly predict compounds’ affinities. MDMB-CHMICA (**13**), which also showed subnanomolar affinities for CB_1_ and CB_2_ receptors, was previously found to be involved in fatal intoxications, and it was concluded that the compound could cause multiple organ failure with lethal outcome in combination with alcohol [39, 40]. The corresponding indazole MDMB-CHMINACA (**14**) again showed even slightly higher affinities for both receptors.

Next, compounds with a valinamide substitution (R^1^) were studied. These were somewhat less potent than the valine methyl esters [compare 5F-AB-PICA (**15**)/MMB-2201 (**7**); AB-CHMINACA (**20**)/MA-CHMINACA (**10**); and 5F-AB-PINACA (**16**)/5F-AMB (**8**)]. 5F-AB-PICA (**15**), a 5F-pentyl-indole derivative, displayed affinities of 35.0 nM and 89.0 nM for CB_1_ and CB_2_ receptors, respectively, while the corresponding indazole 5F-AB-PINACA (**16**) was more potent displaying affinities in the low nanomolar range. We further investigated the 5Cl-pentyl derivative 5Cl-AB-PINACA (**17**), which showed comparable *K*_i_ values to 5F-AB-PINACA (**16**) at 4.06 nM for CB_1_ and 12.0 nM for CB_2_. The *m*-fluorobenzyl and the *o*-fluorobenzyl derivatives (**18** and **19**) showed similar affinities at the CB_1_ receptor, as also previously reported by Buchler et al. [37], with *K*_i_ values in the nanomolar range, and somewhat lower affinity for the CB_2_ receptor. AB-CHMINACA (**20**) displayed low nanomolar CB_1_ and CB_2_ affinity in agreement with previous results by Wiley et al. [41].

5F-ADB-PINACA isomer 2 (**26**) contains a structural isomer of isoleucinamide with a different side chain. This modification resulted in a slight decrease in affinities to CB_1_ and CB_2_ as compared to 5F-ADB-PINACA (**23**), the corresponding *tert*-leucinamide. Furthermore,* tert*-leucinamides, have been investigated which contain a *tert*-butyl group. The 5-fluoropentyl-substituted indole derivative 5F-ADBICA (**21**) showed nanomolar affinities with a *K*_i_ of 2.72 nM at CB_1_ and 1.83 nM at CB_2_ receptors. This was in agreement with data published by Banister et al. [42], who had reported similar EC_50_ values. We found the corresponding indazole derivative **23** to be slightly more potent with *K*_i_ values at 1.43 nM for CB_1_ and 0.694 nM for CB_2_. Banister et al. had determined a higher potency at CB_1_ with an EC_50_ value of 0.24 nM in their membrane potential assay, but a slightly higher EC_50_ value at CB_2_ (2.1 nM). The *p*-fluorobenzyl-substituted indazole ADB-FUBINACA (**24**) showed even lower *K*_i_ values of 0.360 nM for CB_1_ and 0.339 nM for CB_2_. The indole ADB-CHMICA (**22**) was substituted in the R^2^ position with a cyclohexylmethyl residue and showed a *K*_i_ value of 1.24 nM for the CB_1_ and 0.628 nM for the CB_2_ receptor. The corresponding indazole MAB-CHMINACA (**25**), which had been introduced by Buchler et al. [37], was even more potent with a *K*_i_ value of 0.333 nM for CB_1_ and 0.331 nM for CB_2_, which fits well with data reported by Buchler et al. for CB_1_ (no data for CB_2_ had been published by them).

PX-1 (**27**) and PX-2 (**28**) are phenylalaninamide derivatives, PX-1 (**27**) with an indole core and PX-2 (**28**) with an indazole core structure. PX-2 (**28**) showed a *K*_i_ value for the CB_1_ receptor of 127 nM and was thus significantly less potent than the corresponding *tert*-leucinamide derivative 5F-ADB-PINACA (**23**). The *K*_i_ value at CB_2_ (17.4 nM) was also higher than the *K*_i_ value of 0.694 nM determined for 5F-ADB-PINACA (**23**). Indole derivative PX-1 (**27**) displayed a *K*_i_ value of 485 nM for CB_1_, corresponding to a fourfold decrease in affinity as compared to the indazole PX-2 (**28**). The *K*_i_ value at CB_2_ (164 nM) was about tenfold higher. This confirms that the indazole ring system generally leads to a higher affinity as compared to the indole core structure.

APP-FUBINACA (**29**) and APP-CHMINACA (**30**) had been introduced by Buchler et al. [37]. Both are indazoles varying in position R^2^. The *p*-fluorobenzyl derivative APP-FUBINACA (**29**) showed potencies for both CB receptor subtypes of around 50 nM, while the corresponding cyclohexylmethyl derivative APP-CHMINACA (**30**) was more potent displaying *K*_i_ values of 9.81 nM for CB_1_ and 4.39 nM for CB_2_.

Instead of an amino acid residue, the R^1^ position has also been substituted with a cumyl moiety. These types of compounds were first described by Bowden and Williamson [43] and it has recently been found in illicit drug material. For all three investigated cumyl derivatives (**31**–**33**), we could demonstrate affinities in the low nanomolar range for the CB_1_ receptor. Bowden and Williamson had reported subnanomolar EC_50_ values in their functional assays using a homogeneous time-resolved fluorescence (HTRF)-based cAMP assay [43]. The indole derivatives Cumyl-PICA (**31**) and 5F-Cumyl-PICA (**32**) in our hands displayed potencies of around 25 nM for the CB_2_ receptor, while Cumyl-THPINACA (**33**) bearing a 4-tetrahydropyranylmethyl moiety (for R^2^) was more potent with a *K*_i_ value of 1.38 nM at the CB_2_ receptor, which was similar to its *K*_i_ value at the CB_1_ receptor.

The investigated series of compounds contained one member with a 3-oxycarbonyl linker: MO-CHMINACA (**34**), an indazole with a cyclohexylmethyl residue for R^2^ and a methoxycarbonyl-*tert*-leucine for R^1^. It displayed a *K*_i_ value of 10.4 nM at CB_1_ and 1.11 nM at CB_2_ receptors. The only other cyclohexylmethyl-substituted compound investigated by us was BB-22 (see our previous study [25]), which exhibited a *K*_i_ value of 0.217 nM for CB_1_ receptor; however it was substituted with a quinolone for R^1^ and contained an indole core.

Three 3-carbonylindoles (**35**–**37**) were studied. FUB-JWH-018 (**35**), substituted with a naphthyl residue for R^1^ and possessing a *p*-fluorobenzyl residue for R^2^ displayed similarly high nanomolar affinities like the previously studied naphthoyl indazoles THJ018 and THJ2201 [25]. MAM-2201 and EAM-2201, which were substituted with methyl or ethyl in the 4-position of the naphthoyl residue, had shown subnanomolar affinities [25]. Here we report F-2201 (**36**) and Cl-2201 (**37**), the respective 4-fluoro- and 4-chloro derivatives. Both displayed high affinities at 1–2 nM for both CB_1_ and CB_2_ receptors. The previously described alkyl-substituted naphthoyl derivatives had shown similar potencies (compare MAM-2201 and EAM-2201) [25]. The substitutions can be ranked in the following order of potency at CB_1_: ethyl > fluoro > chloro > methyl, while for CB_2_ it was: ethyl > methyl > fluoro ≈ chloro.

The indole derivative mepirapim (**38**) belongs to the 3-amido-substituted derivatives, featuring a 4-methylpiperazinyl residue for R^1^. Mepirapim (**38**) was originally identified by Uchiyama et al. [44] and has been found in "Spice" preparations. We determined an affinity of 2650 nM for the CB_1_ receptor and 1850 nM for the CB_2_ receptor. Therefore, it can be regarded as a rather weak CB receptor ligand.

We further investigated three structurally dissimilar compounds, **39**–**41**, which contain a carbazole core substituted in position 3 with residues typically observed in position R^1^ of indazole- and indole-based compounds. EG-018 (**39**) and EG-2201 (**40**) feature a carbonyl linker connected to a naphthyl residue, whereas MDMB-CHMCZCA (**41**) is substituted with a methoxycarbonyl-*tert*-leucine residue through an amide linker. EG-018 (**39**) displayed low nanomolar affinities with *K*_i_ values of 7.17 nM for CB_1_ and of 2.27 nM for the CB_2_ receptor. EG-018 (**39**) can be compared to JWH-018 (**3**), which showed similar affinities. EG-2201 (**40**) was less potent at CB_1_ with a *K*_i_ value of 22.4 nM, but only slightly more potent at CB_2_ (*K*_i_ = 4.36 nM). MDMB-CHMCZCA (**41**) also displayed affinities in the low nanomolar range. The observed switch from indoles and indazoles to carbazoles can be interpreted as a reaction to the NpSG legislation and similar regulations in other countries that restricted the whole class of indoles and indazoles based on the known SARs. Recently, the synthetic cannabinoid Cumyl-PEGACLONE was identified as one of the first cannabimimetic compounds to circumvent these regulations; it consists of a γ-carboline, another new scaffold for cannabinoid receptor agonists [45]. Carbazoles (**39**–**41**) represent a further new scaffold which circumvents restrictions applied by many, especially European, countries by simply exchanging the well-established bicyclic core structures of indole or indazole for a tricyclic carbazole ring system.

We further investigated the benzimidazole derivative FUBIMINA (**42**), which had previously been described by Wiley et al. [41], and determined a *K*_i_ value of 502 nM at the CB_1_ receptor, which is in the same range as the reported *K*_i_ value of 296 nM, and a *K*_i_ value of 99.0 nM for the CB_2_ receptor, which is slightly higher than the reported value of 23.5 nM [41].

The presently investigated set of compounds complements our previous efforts to study the SARs of synthetic cannabinoids [25]. Of special interest is the observed scaffold hopping. Carbazole derivatives with a high affinity for CB receptors circumvent restriction by current law and display a new lead structure for CB receptor ligands. Further insight into the SARs is required to describe the potency profile of this compound class in more detail.

### cAMP accumulation assays

**Fig. 3:**
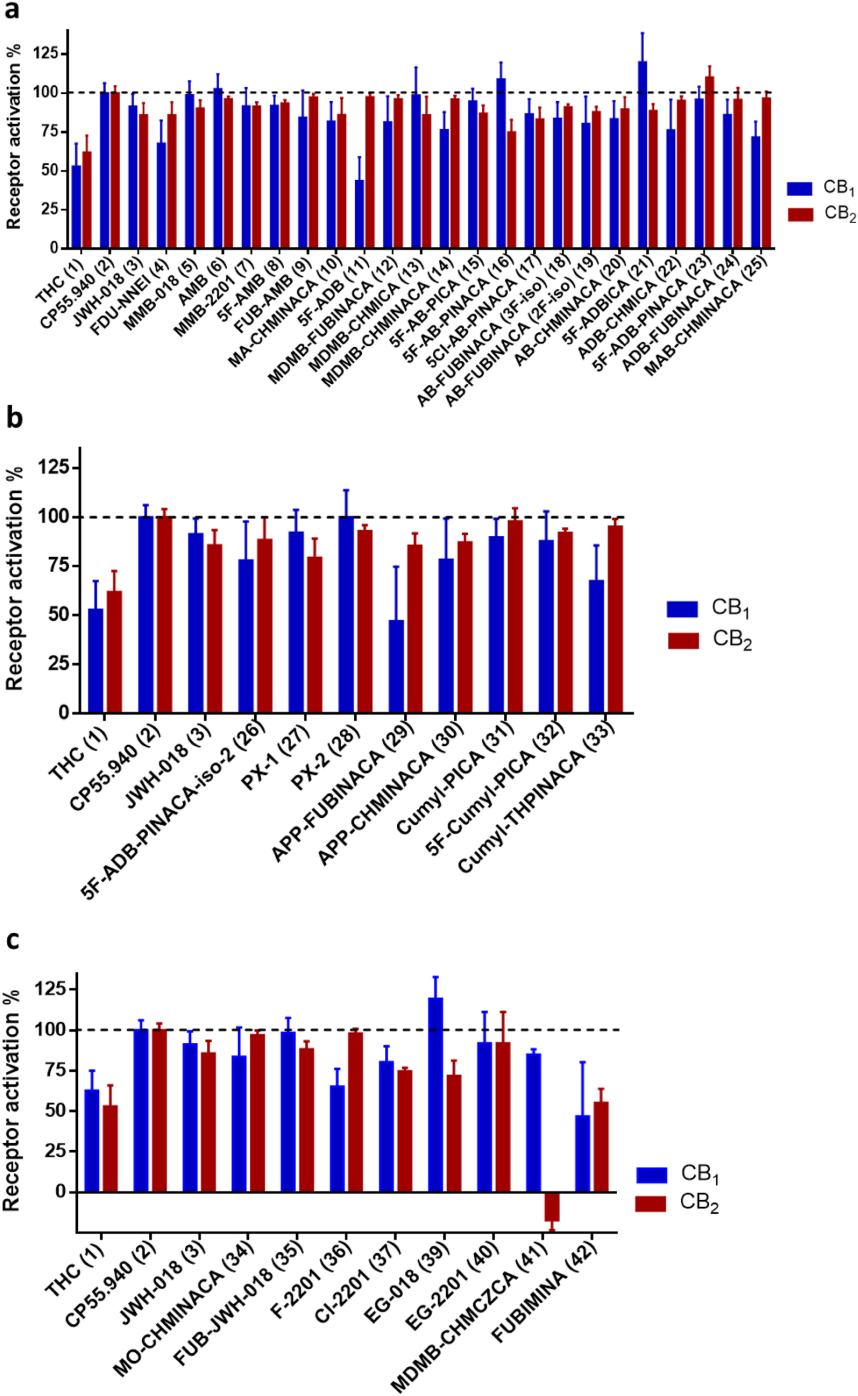
Receptor activation in cAMP accumulation assays. Receptor activation was normalized to the maximal effect observed with the full agonist CP55,940 (1 µM). Compounds were applied at 10 µM concentration in case their *K*_i_ value was ≥ 10 nM and at 1 µM concentration if their *K*_i_ value was < 10 nM. **a** Compounds **4**–**25**; **b** compounds **26**–**33**; **c** compounds **34**–**42**

As a next step, we investigated the compounds in cAMP accumulation assays, to obtain information on their functionality (Fig. 3). CB receptors are G_i_ protein-coupled and thus reduce the levels of cAMP in the cells upon activation. We applied the compounds at either 10 or 1 µM concentration depending on the *K*_i_ values measured in radioligand binding. If the *K*_i_ value was higher than 10 nM, we applied 10 µM of the compound in our assays; otherwise the lower concentration of 1 µM was assumed to be sufficient for maximal CB receptor activation. For comparison, we studied CP55,940 (1 µM), Δ^9^-THC (10 µM), and JWH-018 (1 µM) under the same conditions at concentrations at which they exert their maximal effects. The cAMP response of the full agonist CP55,940 (1 µM) was set at 100% receptor activation.

**Fig. 4:**
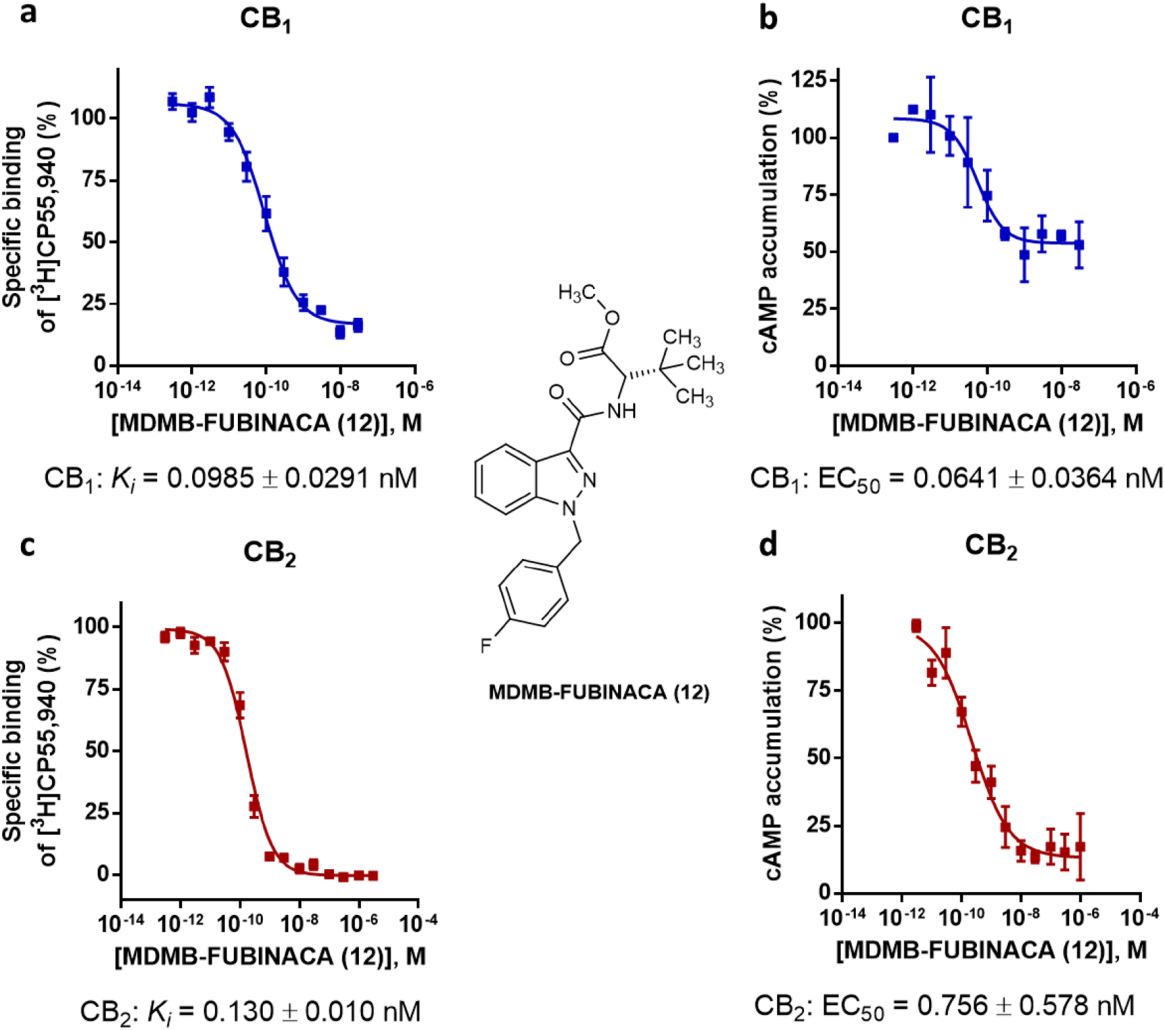
Pharmacological characterization of MDMB-FUBINACA (**12**). **a** Affinity of MDMB-FUBINACA (**12**) for the cannabinoid receptor CB_1_ determined in radioligand binding studies. **b** Receptor activation of the cannabinoid CB_1_ receptor by MDMB-FUBINACA (**12**) determined in cAMP accumulation assays. **c** Affinity of MDMB-FUBINACA (**12**) for the cannabinoid receptor CB_2_ determined in radioligand binding studies. **d** Receptor activation of the cannabinoid CB_2_ receptor by MDMB-FUBINACA (**12**) measured in cAMP accumulation assays

Moreover, we determined the EC_50_ values of MDMB-FUBINACA (**12**) by measuring full concentration inhibition curves. This compound had shown very low *K*_i_ values in radioligand binding assays indicating extremely high affinities, and in fact, the EC_50_ values determined in cAMP assays [EC_50_ values of 0.0641 nM (CB_1_) and 0.756 nM (CB_2_)] were in the same range as the *K*_i_ values measured in binding studies (see Fig. 4).

As can be seen in Fig. 3, almost all of the investigated compounds displayed agonistic behavior and showed high efficacy. Two compounds [5F-ADB (**11**) and APP-FUBINACA (**29**)] displayed a partial activation of the CB_1_ receptor at a concentration of 10 µM. At the CB_2_ receptor however, they were found to act as full agonists (Fig. 3). FUBIMINA (**42**) showed only partial activation of both CB_1_ and CB_2_ receptors at a concentration of 10 µM; the activation was similar to that of Δ^9^-THC, which is a partial agonist. Full receptor activation by FUBIMINA (**42**) might not have been observed, due to its low affinity for the receptors. In accordance with this, Wiley et al. [41] observed a micromolar EC_50_ of 2470 nM in [^35^S]GTPγS assays for FUBIMINA.

**Fig. 5:**
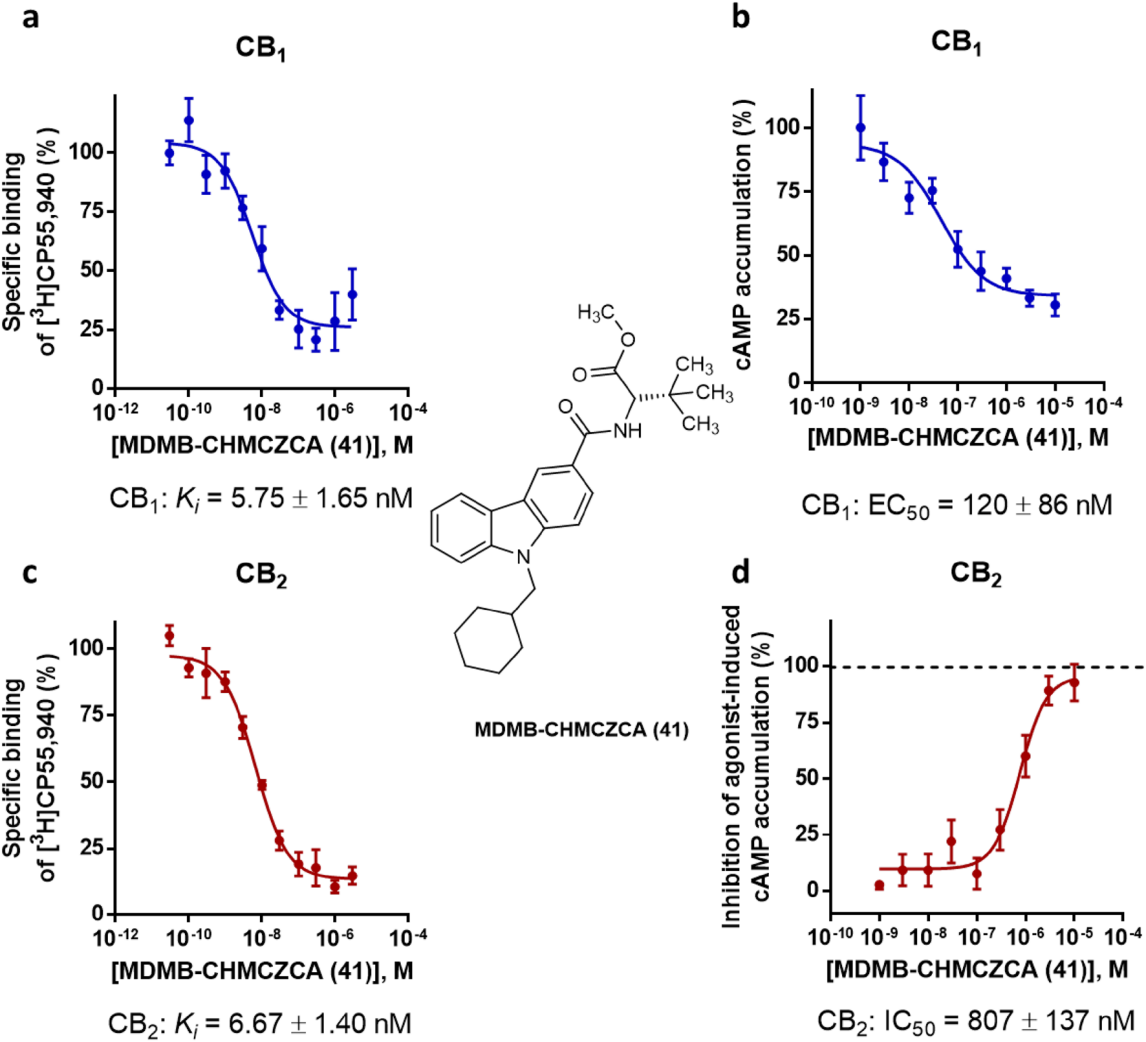
Pharmacological characterization of the carbazole derivative MDMB-CHMCZCA (**41**). **a** Affinity of MDMB-CHMCZCA (**41**) for the cannabinoid receptor CB_1_ determined in radioligand binding studies. **b** Receptor activation of the cannabinoid CB_1_ receptor by MDMB-CHMCZCA (**41**) determined in cAMP accumulation assays. **c** Affinity of MDMB-CHMCZCA (**41**) for the cannabinoid receptor CB_2_ determined in radioligand binding studies. **d** Inhibition of cannabinoid CB_2_ receptor activation induced by CP55,940 (0.03 µM) by MDMB-CHMCZCA (**41**) measured in cAMP accumulation assays

**Fig. 6:**
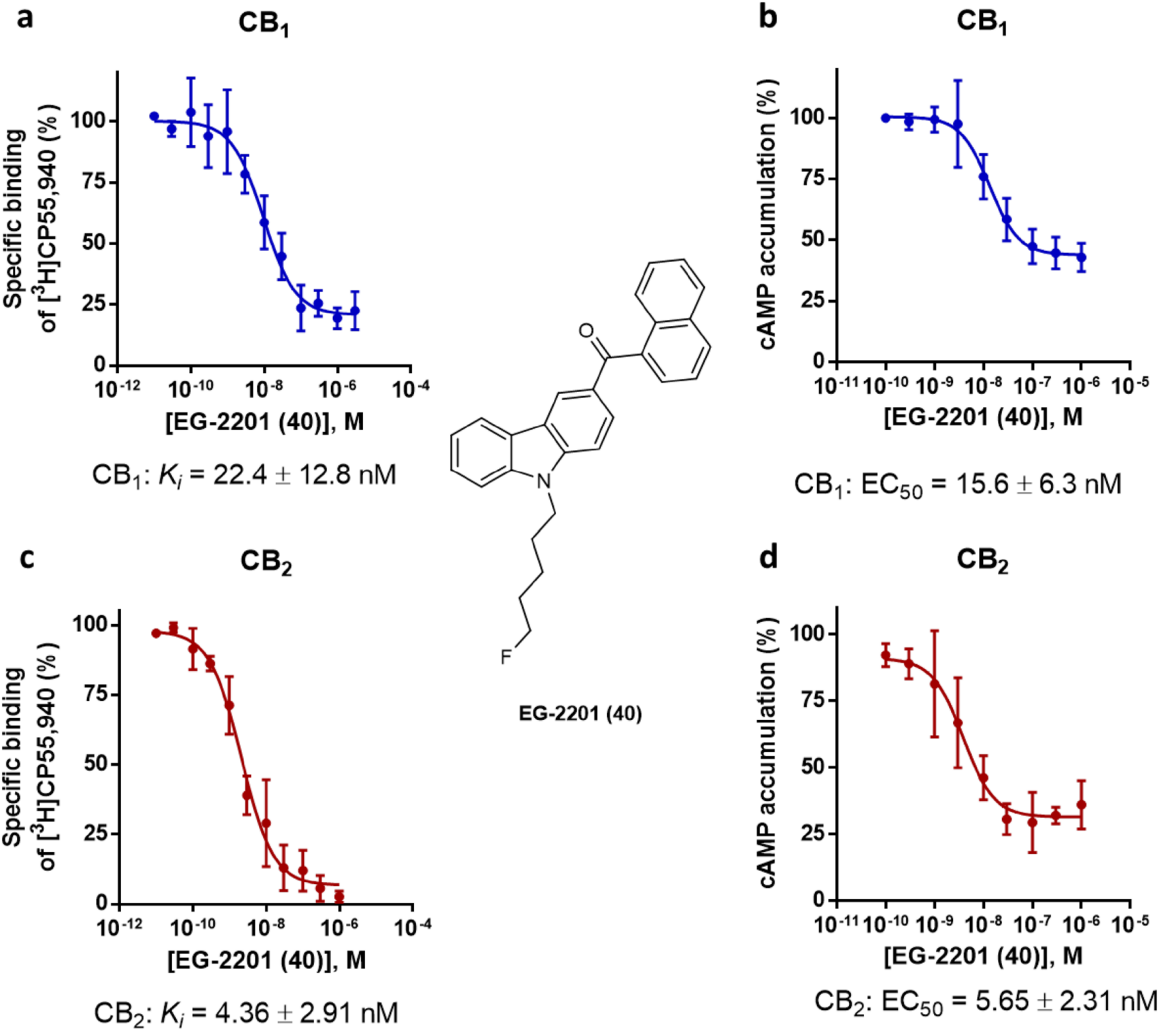
Pharmacological characterization of EG-2201 (**40**). **a** Affinity of EG-2201 (**40**) for the cannabinoid receptor CB_1_ in radioligand binding studies. **b** Receptor activation of the cannabinoid CB_1_ receptor by EG-2201 (**40**) determined in cAMP accumulation assays, as compared to the effect of forskolin (10 µM). **c** Affinity of EG-2201 (**40**) for the cannabinoid receptor CB_2_ determined in radioligand binding studies. **d** Receptor activation of the cannabinoid CB_2_ receptor by EG-2201 (**40**) measured in cAMP accumulation assays, as compared to the effect of forskolin (10 µM)

One compound completely lacked CB_2_ receptor activation: MDMB-CHMCZCA (**41**). However, MDMB-CHMCZCA (**41**) had shown high affinity for the CB_2_ receptor with a *K*_i_ value of 6.67 nM in radioligand binding studies. A higher concentration of MDMB-CHMCZCA (**41**) at 10 µM also failed to evoke an agonistic response (Fig. S3) on the CB_2_ receptor. Therefore, we investigated whether MDMB-CHMCZCA (**41**) might behave as a CB_2_ receptor antagonist. In Fig. 5d, the concentration-dependent response of MDMB-CHMCZCA (**41**) versus CP55,940 as an agonist (0.03 µM corresponding to its EC_80_ value) is shown. MDMB-CHMCZCA (**41**) displayed an IC_50_ value of 807 ± 137 nM under these conditions and clearly behaved as an antagonist at the CB_2_ receptor. The determined IC_50_ value in the cAMP assay was higher than the *K*_i_ value measured in radioligand binding studies. This might be due to the rather high concentration of CP55,940, that was applied, thus underestimating the inhibitory potency of **41**. However, at the CB_1_ receptor MDMB-CHMCZCA (**41**) displayed agonistic behavior (Fig. 5b) with an EC_50_ value of 120 nM and showed full efficacy as compared to the full agonist CP55,940 (Fig. S4). Another carbazole derivative, EG-2201 (**40**) was investigated and found to induce agonistic behavior at both CB receptor subtypes. Its respective *K*_i_ and EC_50_ values were similar (CB_1_ *K*_i_ = 22.4 nM; EC_50_ = 15.6 nM; CB_2_ *K*_i_ = 4.36 nM and EC_50_ = 5.65 nM (see Fig. 6). It showed an efficacy of 94% at CB_1_ and 77% at the CB_2_ receptor as compared to the maximum response of the full agonist CP55,940 (see Fig. S4).

**Fig. 7:**
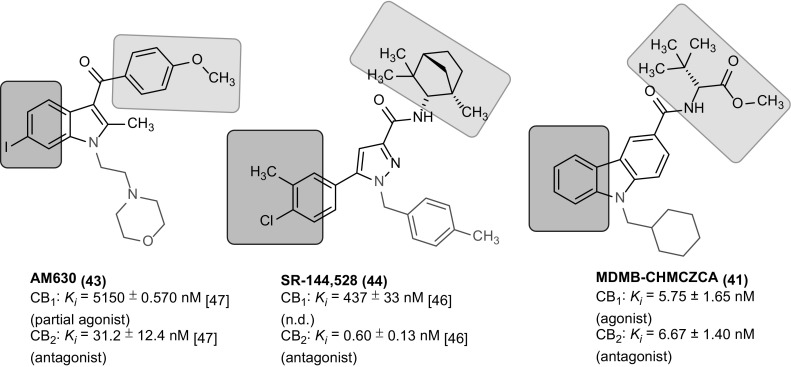
Structural comparison of CB_2_ antagonists/inverse agonists [46, 47]

There are not many CB_2_ receptor antagonists known in the literature. As tool compounds, the inverse agonists AM-630 (**43**), an indole derivative, and SR-144,528 (**44**), a bornyl-substituted pyrazole, structurally related to the CB_1_ receptor inverse agonist rimonabant, are frequently employed. They are both selective for CB_2_ versus CB_1_ [46, 47]. This selectivity for the CB_2_ receptor might primarily be caused by the bulky lipophilic substituent attached to the heterocyclic core (R^1^ position). Compared to these structures (see Fig. 7), MDMB-CHMCZCA (**41**) represents a new class of CB_2_ receptor antagonists. It shares the bulky substitution of the known CB_2_ inverse agonists at R^1^, which covers a similar space as the bornyl substitutent of **44** (Fig. 7). Moreover, AM-630 (**43**) and SR-144,528 (**44**), share bulky lipophilic substituents at position 6 of the indole, or the methyl-chloro-phenyl moiety, respectively. MDMB-CHMCZCA (**41**) resembles these antagonists due to its voluminous tricyclic carbazole structure. While agonists induce a conformational change of the receptors leading to activation, competitive antagonists are often larger than agonists and just block the orthosteric binding site thereby preventing binding of the agonist.

### Molecular docking studies

**Fig. 8:**
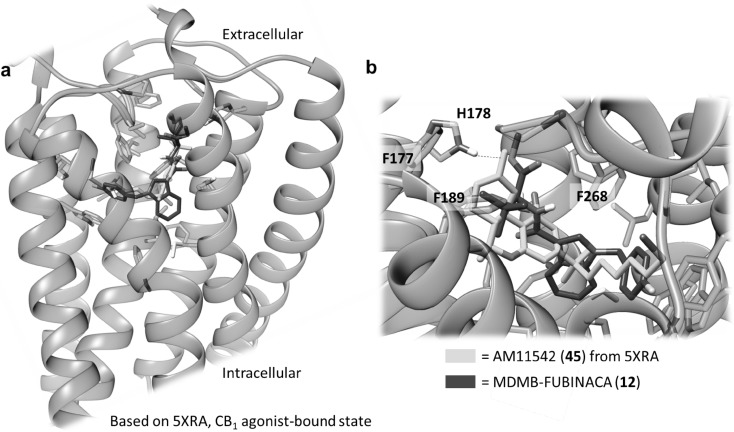
Docking of MDMB-FUBINACA (**12**) into the CB_1_ agonist state crystal structure reveals a plausible binding mode, in which the *p*-fluorobenzyl residue aligns with the alkyl side chain of the Δ^9^-THC-derived co-crystallized AM11542 (**45**, see Fig. 9)

**Fig. 9:**
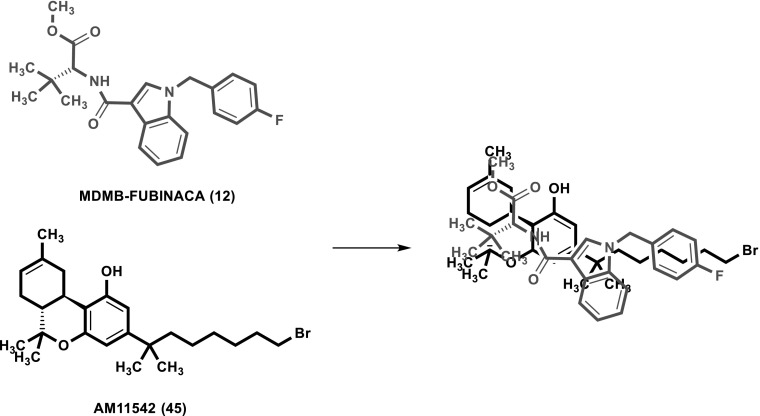
Overlay of the CB_1_ receptor agonists MDMB-FUBINACA (**12**) and AM11542 (**45**)

Recently, the crystal structure of the CB_1_ receptor was determined in both agonist- and antagonist-bound states with resolutions between 2.6 and 2.95 Å [33, 48, 49]. In a molecular docking study, we investigated possible binding poses and interactions of MDMB-FUBINACA (**12**), the most potent CB_1_ agonist of the present series (CB_1_ *K*_i_ = 0.0985 nM). For modeling of its interaction with MDMB-FUBINACA (**12**), we used the agonist-bound template structures. In both published templates, Δ^9^-THC-derived compounds were co-crystallized with the receptor. Here we compare these poses with the hypothetical poses obtained by the docking of MDMB-FUBINACA (**12**). The docking procedure was carried out using the Rosetta protein modeling suite of programs. The binding pose depicted by the largest cluster of low scoring models aligns the *p*-fluorobenzyl residue of MDMB-FUBINACA (**12**) with the alkyl side chain of the Δ^9^-THC derivative AM11542 (**45**) bound in the crystal structure (see Figs. 8, 9). This pose is regarded as plausible because the length of the *p*-fluorobenzyl residue of MDMB-FUBINACA (**12**) is of importance for CB_1_-selectivity versus CB_2_ and closely resembles the lipophilic side chain of **45**. The co-crystallized agonist **45** showed a *K*_i_ value of 0.11 nM for the CB_1_ receptor [33], which is very similar to the affinity of MDMB-FUBINACA (**12**, *K*_*i*_ 0.0985 nM). As shown in Figs. 8 and 9, the shape and size of both agonists as well as their lipophilicity and potential types of interaction aligned quite well. However, the template shows an interaction of serine-383 as a hydrogen bond donor to the phenolic group of the Δ^9^-THC-like compound. This was not observed in our model. Instead the oxygen atom of the ester function may participate in a hydrogen bond with histidine-178, an interaction that was not found for the co-crystallized compounds but could explain the equally high affinity of MDMB-FUBINACA (**12**) to the CB_1_ receptor observed in the present study. A plausible structural overlay of Δ^9^-THC derivative AM11542 (**45**) and MDMB-FUBINACA (**12**) is depicted in Fig. 9. Alternative binding poses were less often sampled and showed a superimposition of the *tert*-leucine methyl ester residue with the alkyl side chain (compare Fig. S1).

### Potency at the orphan cannabinoid-interacting GPCRs GPR18 and GPR55

The orphan receptors GPR18 and GPR55 can interact with certain natural and synthetic cannabinoids [26–29]. Recently, we found that some "Spice" constituents behaved as weak GPR55 antagonists [25]. Therefore, we investigated the new series of indole, indazole, benzimidazole and carbazole-derived structures in β-arrestin assays at GPR18 and GPR55 (Table 2). Most of the compounds were inactive. At GPR55, Cl-2201 (**37**) showed the highest antagonistic potency, tested versus the GPR55 agonist lysophosphatidylinositol (LPI, 1 µM), displaying an IC_50_ value of 7.12 µM. The fluorinated analogue F-2201 (**36**) was somewhat less potent with an IC_50_ value of 22.1 µM. Both of these compounds are derivatives of EAM-2201, which in our previous study had shown an IC_50_ value of 1.86 µM [25]. For the lipophilic substitutions, the following rank order of potency was observed: ethyl > methyl > chloro > fluoro. Therefore, it can be concluded that a lipophilic substitution in position 4 of the naphthyl residue was a requirement for GPR55 inhibition. The first amino acid-substituted derivatives to act as GPR55 antagonists are MO-CHMINACA (**34**) with an IC_50_ value of 9.29 µM and MDMB-CHMINACA (**14**) with an IC_50_ value of 10.3 µM. At GPR18 weak inhibitory potency was observed for MDMB-CHMICA (**13**), MO-CHMINACA (**34**) and MDMB-CHMCZCA (**41**).

**Table 2:** Activity of test compounds in β-arrestin assays at human GPR55 and GPR18

	Compd	Human GPR55		Human GPR18	
		EC_50_ (µM) (% activation)	IC_50_ (µM) (% inhibition)	EC_50_ (µM) (% activation)	IC_50_ (µM) (% inhibition)
**1**	Δ^9^-THC	–	14.2 [52]	4.61 [52]	–
**2**	CP55,940	–	1.61 [53]	–	5.99 [52]
3-Amido-indole and -indazoles (**A**)					
**4**	FDU-NNEI	> 10 (13%)	> 10 (2%)	> 10 (20%)	> 10 (− 1%)
**5**	MMB-018	> 10 (5%)	> 10 (5%)	> 10 (13%)	> 10 (− 4%)
**6**	AMB	> 10 (15%)	> 10 (28%)	> 10 (42%) (n = 1)	> 10 (15%)
**7**	MMB-2201	> 10 (5%)	> 10 (− 10%)	> 10 (2%)	> 10 (− 6%)
**8**	5F-AMB	> 10 (14%)	> 10 (− 6%)	> 10 (1%)	> 10 (− 5%)
**9**	FUB-AMB	> 10 (4%)	> 10 (17%)	> 10 (− 2%)	> 10 (11%)
**10**	MA-CHMINACA	> 10 (2%)	> 10 (31%)	> 10 (19%)	> 10 (10%)
**11**	5F-ADB	> 10 (0%)	> 10 (6%)	> 10 (10%)	> 10 (− 10%)
**12**	MDMB-FUBINACA	> 10 (− 4%)	> 10 (30%)	> 10 (18%)	> 10 (31%)
**13**	MDMB-CHMICA	> 10 (5%)	> 10 (38%)	> 10 (5%)	14.1 ± 3.1^a^
**14**	MDMB-CHMINACA	> 10 (− 5%)	10.3 ± 1.7	> 10 (27%)	≈ 10 (51%)
**15**	5F-ABPICA	> 10 (10%)	> 10 (− 6%)	> 10 (23%)	> 10 (− 25%)
**16**	5F-AB-PINACA	> 10 (15%)	> 10 (− 3%)	> 10 (12%)	> 10 (10%)
**17**	5Cl-AB-PINACA	> 10 (17%)	> 10 (− 8%)	> 10 (4%)	> 10 (0%)
**18**	AB-FUBINACA (3F-benzyl-isomer)	> 10 (11%)	> 10 (− 7%)	> 10 (10%)	> 10 (− 11%)
**19**	AB-FUBINACA (2F-benzyl-isomer)	> 10 (15%)	> 10 (− 9%)	> 10 (11%)	> 10 (− 5%)
**20**	AB-CHMINACA	> 10 (8%)	> 10 (− 3%)	> 10 (13%)	> 10 (11%)
**21**	5F-ADBICA	> 10 (8%)	> 10 (− 8%)	> 10 (27%)	> 10 (− 11%)
**22**	ADB-CHMICA	> 10 (17%)	> 10 (8%)	> 10 (8%)	> 10 (14%)
**23**	5F-ADB-PINACA	> 10 (18%)	> 10 (− 2%)	> 10 (9%)	> 10 (21%)
**24**	ADB-FUBINACA	> 10 (7%)	> 10 (− 3%)	> 10 (7%)	> 10 (16%)
**25**	MAB-CHMINACA	> 10 (16%)	> 10 (− 7%)	> 10 (6%)	> 10 (10%)
**26**	5F-ADB-PINACA-isomer 2	> 10 (2%)	> 10 (− 6%)	> 10 (8%)	> 10 (− 11%)
**27**	PX-1	> 10 (6%)	> 10 (1%)	> 10 (15%)	> 10 (− 16%)
**28**	PX-2	> 10 (16%)	> 10 (− 10%)	> 10 (− 4%)	> 10 (15%)
**29**	APP-FUBINACA	> 10 (26%)	> 10 (− 9%)	> 10 (4%)	> 10 (21%)
**30**	APP-CHMINACA	> 10 (11%)	> 10 (9%)	> 10 (5%)	≈ 10 (57%)
**31**	Cumyl-PICA	> 10 (11%)	> 10 (3%)	> 10 (19%)	> 10 (− 7%)
**32**	5F-Cumyl-PICA	> 10 (14%)	> 10 (− 6%)	> 10 (16%)	> 10 (− 3%)
**33**	Cumyl-THPINACA	> 10 (11%)	> 10 (7%)	> 10 (11%)	> 10 (9%)
Ester-substituted indazoles (**B**)					
**34**	MO-CHMINACA	> 10 (1%)	9.29 ± 1.7	> 10 (0%)	12.6 ± 3.5^a^
3-Carbonyl-indoles (**C**)					
**35**	FUB-JWH-018	> 10 (7%)	> 10 (30%)	> 10 (20%)	> 10 (30%)
**36**	F-2201	> 10 (− 10%)	22.1 ± 12.2^a^	> 10 (18%)	> 10 (14%)
**37**	Cl-2201	> 10 (− 1%)	7.12 ± 1.26	> 10 (16%)	> 10 (4%)
3-Carbonyl-carbazoles (**E**)					
**39**	EG-018	> 10 (0%)^b^	> 10 (1%)^b^	> 10 (2%)^b^	> 10 (− 11%)
**40**	EG-2201	> 10 (− 3%)	> 10 (3%)	> 10 (3%)	> 10 (7%)
**41**	MDMB-CHMCZCA	> 10 (− 8%)	> 10 (25%)	> 10 (− 35%)	9.66 ± 1.20^a^
Carbonyl-benzimidazole (**F**)					
**42**	FUBIMINA	> 10 (16%)	> 10 (44%)	> 10 (42%)^c^	>10 (14%)^b^

^a^Extrapolated values; full curve could not be determined due to limited solubility

^b^*n *= 2

^c^*n *= 1

These results indicate that the investigated series of CB receptor ligands is highly selective versus GPR18 and GPR55. None of the compounds was able to activate these orphan receptors. Some acted as antagonists at micromolar concentrations, but considerable efforts would be required to optimize these new lead structures to obtain potent GPR18- or GPR55-selective antagonists.

## Conclusions

In this study, we continued to investigate the SARs of illicitly used constituents of "Spice" preparations. We investigated the affinities of a large series of compounds in radioligand binding assays and found MDMB-FUBINACA (**12**) belonging to the class of 3-amidoindazoles to be an extremely potent fully efficacious agonist showing picomolar affinities for CB_1_ (98.5 pM) and CB_2_ (130 pM) receptors. For this compound class severe side effects had been reported, as for example the “zombie outbreak” that was related to AMB-FUBINACA [50], a structurally related compound. The extremely high potency of these compounds might be one of the reasons for their severe side effects. The SARs, especially regarding the R^2^ residue, were consistent with the patterns observed in our previous study [25]. Lipophilic substituents had been introduced, e.g., a 5-fluoropentyl side chain, or a *p*-fluorobenzyl residue, which had similar properties as the pentyl side chain found in the JWH-compounds such as JWH-018. For MDMB-FUBINACA (**12**), we performed CB_1_ receptor docking studies and observed a pose comparable to Δ^9^-THC-derived compounds. In addition to the well-described group of alkylindoles and indazoles, we investigated a series of carbazoles, which showed single-digit nanomolar affinity at both CB receptor subtypes. One of these compounds, MDMB-CHMCZCA (**41**), unexpectedly turned out to be a full agonist at the CB_1_, but an antagonist at CB_2_ receptors, with *K*_*i*_ values at CB_1_ of 5.75 nM and at CB_2_ of 6.67 nM, and EC_50_ values of 120 nM at CB_1_ and of 807 nM at CB_2_ receptors in cAMP accumulation assays. According to our knowledge, this combination of full CB_1_-agonistic and CB_2_-antagonistic activities is unique. Although CB_2_ receptor antagonists and inverse agonists have been studied for some time, their clinical utility is still under investigation. The expression of CB_2_ receptors in the immune system suggests immunomodulatory effects for CB_2_ receptor ligands. The group of carbazoles showed nanomolar affinities for the CB_1_ receptor and behaved as full agonists in cAMP accumulation assays. They circumvent the structural features described in the NpSG by scaffold hopping. This new class of synthetic cannabinoids needs to be further studied to fully investigate its SARs and potential for abuse. The present study may contribute to guiding future decisions on the restriction of carbazole-derived and related synthetic cannabinoids.

## Electronic supplementary material

Below is the link to the electronic supplementary material.

Supplementary material 1 (PDF 401 kb)
