# Supplementary material for: Pharmacological evaluation of new constituents of “Spice”: synthetic cannabinoids based on indole, indazole, benzimidazole and carbazole scaffolds
Source: Forensic Toxicol. 2018 Apr 26;36(2):385–403. doi: 10.1007/s11419-018-0415-z (PMC6002460; doi:10.1007/s11419-018-0415-z)
Supplement: Supplementary file 1 — Supplementary material 1 (PDF 401 kb) [file 11419_2018_415_MOESM1_ESM.pdf]

Pharmacological evaluation of new constituents of "SPICE" – synthetic cannabinoids based on indole, indazole, benzimidazole and carbazole scaffolds

Clara T. Schoeder,[1,3] Cornelius Hess,[2] Burkhard Madea,[2] Jens Meiler,[4] Christa E. Müller[1,3]

[1] PharmaCenter Bonn, Pharmaceutical Institute, Pharmaceutical Chemistry I, University of Bonn, An der Immenburg 4, 53121 Bonn, Germany

[2] Institute of Forensic Medicine, Forensic Toxicology, University Hospital of Bonn, Stiftsplatz 12, 53111 Bonn, Germany

[3] Research Training Group 1873, University of Bonn, 53127 Bonn, Germany

[4] Departments of Chemistry and Pharmacology, Vanderbilt University, Stevenson Center, Station B 351822, Nashville, TN 37235, USA

*Address correspondence to:
Prof. Dr. Christa Müller
Pharmazeutisches Institut, Pharmazeutische Chemie I
An der Immenburg 4, D-53121 Bonn, Germany
Phone: +49-228-73-2301
Fax: +49-228-73-2567
E-Mail: christa.mueller@uni-bonn.de

Content | **Page**

Docking procedure S3

Figure S1: ligand RMSD versus Score (interface delta) S9

Figure S2: Poses obtained from docking. A and C show a pose, where the N-valine methyl ester aligns with the alkyl side chain of the THC-derivative, whereas B and D show poses, in which the p-fluorobenzyl residue aligns with the alkyl side chain of the THC-derivative. A and B are derived from 5xr8 and C and D from 5xra. Green = AM841/AM11542 (**45**), blue = MDMB-FUBINACA (**12**) S10

Figure S3: Cannabinoid receptor activation by MDMB-CHMCZCA (**41**) determined in cAMP accumulation assays S11

Figure S4: Receptor activation of the cannabinoid receptor CB_1 (**A**-**C**) and CB_2 (**D**-**E**) by MDMB-FUBINACA (**12**, **A**, **D**), EG-2201 (**40**, **B**, **D**) and MDMD-CHMCZCA (**41**, **C**). **F.** Efficacy of tested compounds compared to max. response of CP55,940 (**2**) S12

Docking procedure

The docking procedure was carried out with RosettaLigand (www.RosettaCommons.org) using the 2017.08.59291 build) [1],[2].

The ligand was processed to a Rosetta-friendly format resulting in a library of conformers.

```
NAME LG1
IO_STRING LG1 Z
TYPE LIGAND
AA UNK
ATOM  C8  aroC  X   -0.07
ATOM  C5  aroC  X   -0.07
ATOM  C4  aroC  X   -0.07
ATOM  C3  aroC  X   -0.07
ATOM  C2  aroC  X   -0.07
ATOM  C1  aroC  X   -0.07
ATOM  C6  aroC  X   -0.07
ATOM  H4  Haro  X   0.16
ATOM  H1  Haro  X   0.16
ATOM  H2  Haro  X   0.16
ATOM  H3  Haro  X   0.16
ATOM  N1  Nhis  X   -0.49
ATOM  C7  aroC  X   -0.07
ATOM  H5  Haro  X   0.16
ATOM  C10 CH2   X   -0.14
ATOM  C11 aroC  X   -0.07
ATOM  C12 aroC  X   -0.07
ATOM  C13 aroC  X   -0.07
ATOM  C14 aroC  X   -0.07
ATOM  C15 aroC  X   -0.07
ATOM  C16 aroC  X   -0.07
ATOM  H12 Haro  X   0.16
ATOM  H11 Haro  X   0.16
ATOM  F1  F     X   -0.21
ATOM  H10 Haro  X   0.16
ATOM  H9  Haro  X   0.16
ATOM  H7  Hapo  X   0.14
ATOM  H8  Hapo  X   0.14
ATOM  C9  COO   X   0.66
ATOM  O1  ONH2  X   -0.51
ATOM  N2  Ntrp  X   -0.57
ATOM  C17 CH1   X   -0.05
ATOM  C18 COO   X   0.66
ATOM  O2  OOC   X   -0.72
ATOM  O3  OH    X   -0.62
ATOM  C20 CH3   X   -0.23
ATOM  H14 Hapo  X   0.14
ATOM  H15 Hapo  X   0.14
ATOM  H16 Hapo  X   0.14
ATOM  C19 CH1   X   -0.05
ATOM  C21 CH3   X   -0.23
ATOM  H17 Hapo  X   0.14
```

```
ATOM  H18 Hapo  X   0.14
ATOM  H19 Hapo  X   0.14
ATOM  C22 CH3   X   -0.23
ATOM  H20 Hapo  X   0.14
ATOM  H21 Hapo  X   0.14
ATOM  H22 Hapo  X   0.14
ATOM  C23 CH3   X   -0.23
ATOM  H23 Hapo  X   0.14
ATOM  H24 Hapo  X   0.14
ATOM  H25 Hapo  X   0.14
ATOM  H13 Hapo  X   0.14
ATOM  H6  Hpol  X   0.47
BOND_TYPE  C1   C2  2
BOND_TYPE  C1   C6  1
BOND_TYPE  C1   H1  1
BOND_TYPE  C2   C3  1
BOND_TYPE  C2   H2  1
BOND_TYPE  C3   C4  2
BOND_TYPE  C3   H3  1
BOND_TYPE  C4   C5  1
BOND_TYPE  C4   N1  1
BOND_TYPE  C5   C6  2
BOND_TYPE  C5   C8  1
BOND_TYPE  C6   H4  1
BOND_TYPE  N1   C7  1
BOND_TYPE  N1   C10 1
BOND_TYPE  C7   C8  2
BOND_TYPE  C7   H5  1
BOND_TYPE  C8   C9  1
BOND_TYPE  C9   O1  2
BOND_TYPE  C9   N2  1
BOND_TYPE  N2   C17 1
BOND_TYPE  N2   H6  1
BOND_TYPE  C10  C11 1
BOND_TYPE  C10  H7  1
BOND_TYPE  C10  H8  1
BOND_TYPE  C11  C12 1
BOND_TYPE  C11  C16 2
BOND_TYPE  C12  C13 2
BOND_TYPE  C12  H9  1
BOND_TYPE  C13  C14 1
BOND_TYPE  C13  H10 1
BOND_TYPE  C14  C15 2
BOND_TYPE  C14  F1  1
BOND_TYPE  C15  C16 1
BOND_TYPE  C15  H11 1
BOND_TYPE  C16  H12 1
BOND_TYPE  C17  C18 1
BOND_TYPE  C17  C19 1
BOND_TYPE  C17  H13 1
BOND_TYPE  C18  O2  2
BOND_TYPE  C18  O3  1
BOND_TYPE  O3   C20 1
BOND_TYPE  C19  C21 1
BOND_TYPE  C19  C22 1
BOND_TYPE  C19  C23 1
BOND_TYPE  C20  H14 1
BOND_TYPE  C20  H15 1
```

```
BOND_TYPE  C20  H16 1
BOND_TYPE  C21  H17 1
BOND_TYPE  C21  H18 1
BOND_TYPE  C21  H19 1
BOND_TYPE  C22  H20 1
BOND_TYPE  C22  H21 1
BOND_TYPE  C22  H22 1
BOND_TYPE  C23  H23 1
BOND_TYPE  C23  H24 1
BOND_TYPE  C23  H25 1
CHI 1  C4   N1   C10  C11
CHI 2  C5   C8   C9   O1
CHI 3  C8   C9   N2   C17
CHI 4  C9   N2   C17  C18
CHI 5  N1   C10  C11  C12
CHI 6  N2   C17  C18  O2
CHI 7  N2   C17  C19  C21
CHI 8  C17  C18  O3   C20
NBR_ATOM  C8
NBR_RADIUS 9.397383
ICOOR_INTERNAL    C8     0.000000    0.000000    0.000000   C8    C5    C4
ICOOR_INTERNAL    C5     0.000000  180.000000    1.413739   C8    C5    C4
ICOOR_INTERNAL    C4     0.000000   72.939828    1.433449   C5    C8    C4
ICOOR_INTERNAL    C3  -178.081945   58.968142    1.411208   C4    C5    C8
ICOOR_INTERNAL    C2    -1.302666   61.098747    1.407972   C3    C4    C5
ICOOR_INTERNAL    C1    -0.547979   58.997918    1.419139   C2    C3    C4
ICOOR_INTERNAL    C6     1.603517   59.964650    1.416310   C1    C2    C3
ICOOR_INTERNAL    H4   179.208937   59.887064    1.049956   C6    C1    C2
ICOOR_INTERNAL    H1  -179.997458   60.015271    1.049981   C1    C2    C6
ICOOR_INTERNAL    H2   179.999934   60.503107    1.049931   C2    C3    C1
ICOOR_INTERNAL    H3  -179.997727   59.451276    1.050009   C3    C4    C2
ICOOR_INTERNAL    N1   179.168649   73.002410    1.386435   C4    C5    C3
ICOOR_INTERNAL    C7    -1.327184   69.939713    1.348030   N1    C4    C5
ICOOR_INTERNAL    H5  -178.953852   54.596664    1.049978   C7    N1    C4
ICOOR_INTERNAL    C10 -176.418950   57.096615    1.447124   N1    C4    C7
ICOOR_INTERNAL    C11  -96.207296   65.840773    1.495939   C10   N1    C4
ICOOR_INTERNAL    C12   59.995416   52.094753    1.425596   C11   C10   N1
ICOOR_INTERNAL    C13  179.870501   60.942328    1.421018   C12   C11   C10
ICOOR_INTERNAL    C14    0.229438   59.880230    1.416734   C13   C12   C11
ICOOR_INTERNAL    C15   -1.268820   61.119116    1.421449   C14   C13   C12
ICOOR_INTERNAL    C16    1.619328   59.405843    1.397668   C15   C14   C13
ICOOR_INTERNAL    H12  179.097267   60.350328    1.050067   C16   C15   C14
ICOOR_INTERNAL    H11  179.996678   60.294620    1.050028   C15   C14   C16
ICOOR_INTERNAL    F1  -179.992135   59.439727    1.376342   C14   C13   C15
ICOOR_INTERNAL    H10  179.998995   60.061642    1.050062   C13   C12   C14
ICOOR_INTERNAL    H9  -179.999966   59.535425    1.049992   C12   C11   C13
ICOOR_INTERNAL    H7  -120.678958   71.717642    1.070047   C10   N1    C11
ICOOR_INTERNAL    H8  -121.665587   73.272279    1.070020   C10   N1    H7
ICOOR_INTERNAL    C9   179.523654   53.338482    1.471068   C8    C5    C4
ICOOR_INTERNAL    O1    -0.995463   53.335989    1.230533   C9    C8    C5
ICOOR_INTERNAL    N2   179.993378   63.330819    1.390685   C9    C8    O1
ICOOR_INTERNAL    C17  179.000772   56.770867    1.478336   N2    C9    C8
ICOOR_INTERNAL    C18 -116.570422   71.646858    1.480140   C17   N2    C9
ICOOR_INTERNAL    O2   -55.994104   54.046965    1.247846   C18   C17   N2
ICOOR_INTERNAL    O3   179.993126   62.975686    1.383005   C18   C17   O2
ICOOR_INTERNAL    C20 -179.998373   63.636285    1.431835   O3    C18   C17
ICOOR_INTERNAL    H14  -79.771489   70.539106    1.069993   C20   O3    C18
ICOOR_INTERNAL    H15  119.989753   70.534311    1.070028   C20   O3    H14
```

```
ICOOR_INTERNAL    H16  119.999972   70.513618    1.069984   C20   O3    H15
ICOOR_INTERNAL    C19 -126.424534   75.699428    1.597209   C17   N2    C18
ICOOR_INTERNAL    C21  -62.003856   64.152627    1.552813   C19   C17   N2
ICOOR_INTERNAL    H17  -55.816744   70.541476    1.069909   C21   C19   C17
ICOOR_INTERNAL    H18  119.987088   70.534845    1.069978   C21   C19   H17
ICOOR_INTERNAL    H19  119.993711   70.517646    1.070062   C21   C19   H18
ICOOR_INTERNAL    C22  121.476753   72.083519    1.519903   C19   C17   C21
ICOOR_INTERNAL    H20  171.206836   70.500562    1.070024   C22   C19   C17
ICOOR_INTERNAL    H21  119.992838   70.541686    1.069982   C22   C19   H20
ICOOR_INTERNAL    H22  120.018321   70.545348    1.070027   C22   C19   H21
ICOOR_INTERNAL    C23  118.401404   68.277953    1.557897   C19   C17   C22
ICOOR_INTERNAL    H23 -174.644420   70.495385    1.070033   C23   C19   C17
ICOOR_INTERNAL    H24  119.990850   70.542533    1.069993   C23   C19   H23
ICOOR_INTERNAL    H25  120.012181   70.540292    1.069980   C23   C19   H24
ICOOR_INTERNAL    H13 -117.960247   63.125624    1.069927   C17   N2    C19
ICOOR_INTERNAL    H6  -179.995566   61.613699    1.020022   N2    C9    C17
PDB_ROTAMERS /home/clary/Dokumente/cannabinoid_modeling/LG_confs.pdb
```

The docking procedure was carried out using the following options and the following docking script.

Docking options

```
-database
/home/clary/Dokumente/rosetta/rosetta_bin_linux_2017.08.59291_bundle/ma
in/database/
-in
  -file
      -extra_res_fa LG.params
-packing
 -ex1
 -ex1aro
 -ex2
-parser
 -protocol new_dock_MDMB.xml

-out
 -file
  -fullatom
 -pdb

-score
```

Docking script

```
<ROSETTASCRIPTS>
	<SCOREFXNS>
		<ligand_soft_rep weights=ligand_soft_rep>
			<Reweight scoretype=fa_elec weight=0.42/>
			<Reweight scoretype=hbond_bb_sc weight=1.3/>
			<Reweight scoretype=hbond_sc weight=1.3/>
			<Reweight scoretype=rama weight=0.2/>
		</ligand_soft_rep>
		<hard_rep weights=ligand>
			<Reweight scoretype=fa_intra_rep weight=0.004/>
			<Reweight scoretype=fa_elec weight=0.42/>
			<Reweight scoretype=hbond_bb_sc weight=1.3/>
			<Reweight scoretype=hbond_sc weight=1.3/>
			<Reweight scoretype=rama weight=0.2/>
		</hard_rep>
	</SCOREFXNS>
	<LIGAND_AREAS>
		<docking_sidechain_X chain=X cutoff=6.0 add_nbr_radius=true
all_atom_mode=true minimize_ligand=10/>
			<final_sidechain_X chain=X cutoff=6.0
add_nbr_radius=true all_atom_mode=true/>
			<final_backbone_X chain=X cutoff=7.0
add_nbr_radius=false all_atom_mode=true Calpha_restraints=0.3/>
	</LIGAND_AREAS>

	<INTERFACE_BUILDERS>
		<side_chain_for_docking ligand_areas=docking_sidechain_X/>
		<side_chain_for_final ligand_areas=final_sidechain_X/>
		<backbone ligand_areas=final_backbone_X extension_window=3/>
	</INTERFACE_BUILDERS>
	<MOVEMAP_BUILDERS>
		<docking sc_interface=side_chain_for_docking
minimize_water=true/>
		<final sc_interface=side_chain_for_final
bb_interface=backbone minimize_water=true/>
	</MOVEMAP_BUILDERS>
	<MOVERS>
		<CompoundTranslate name=compound_translate
randomize_order=false allow_overlap=false>
			<Translate chain=X distribution=uniform angstroms=0.5
cycles=50/>
		</CompoundTranslate>
		<Rotate name=rotate_X chain=X distribution=uniform
degrees=45 cycles=500/>
		<SlideTogether name=slide_together chains=X/>
		<HighResDocker name=high_res_docker cycles=6
repack_every_Nth=3 scorefxn=ligand_soft_rep movemap_builder=docking/>
		<FinalMinimizer name=final scorefxn=hard_rep
movemap_builder=final/>
```

```
            <InterfaceScoreCalculator name=add_scores chains=X
scorefxn=hard_rep/>
            <ParsedProtocol name=low_res_dock>
                  <Add mover_name=compound_translate/>
                  <Add mover_name=rotate_X/>
                  <Add mover_name=slide_together/>
            </ParsedProtocol>
            <ParsedProtocol name=high_res_dock>
                  <Add mover_name=high_res_docker/>
                  <Add mover_name=final/>
            </ParsedProtocol>
            <ddG name=calculateDDG jump=1 per_residue_ddg=1
repack_bound=0 repack_unbound=0 scorefxn=hard_rep/>
      </MOVERS>
      <PROTOCOLS>
            <Add mover_name=low_res_dock/>
            <Add mover_name=high_res_dock/>
            <Add mover_name=add_scores/>
            <Add mover_name=calculateDDG/>
      </PROTOCOLS>
</ROSETTASCRIPTS>
```

Docking command

```
/home/clary/Dokumente/rosetta/rosetta_bin_linux_2017.08.59291_bundle/ma
in/source/bin/rosetta_scripts.default @dock_MDMB.options –l liste.ls –
out:prefix MDMB_ -nstruct 4
```

Models were evaluated by calculating their interface_delta (bound-unbound energy state). This was accomplished by using the InterfaceAnalyzer:

```
/home/clary/Dokumente/rosetta/rosetta_bin_linux_2017.08.59291_bundle/ma
in/source/bin/InterfaceAnalyzer.default @analyze_interface_MDMB.options
–l liste.ls
```

InterfaceAnalyzer options:

```
-in:file:extra_res_fa LG.params
-out:file:score_only interface_scores.fasc
-fixedchains A
-tracer_data_print false
```

Models were analyzed regarding their energetic scoring (interface_delta) and their spacial arrangement using profit [3] for aligning all models and the Biochemical Library (BCL) (http://www.meilerlab.org/index.php/bclcommons/show/b_apps_id/1) for cluster analysis. From different clusters the best scoring models were selected and visually evaluated.

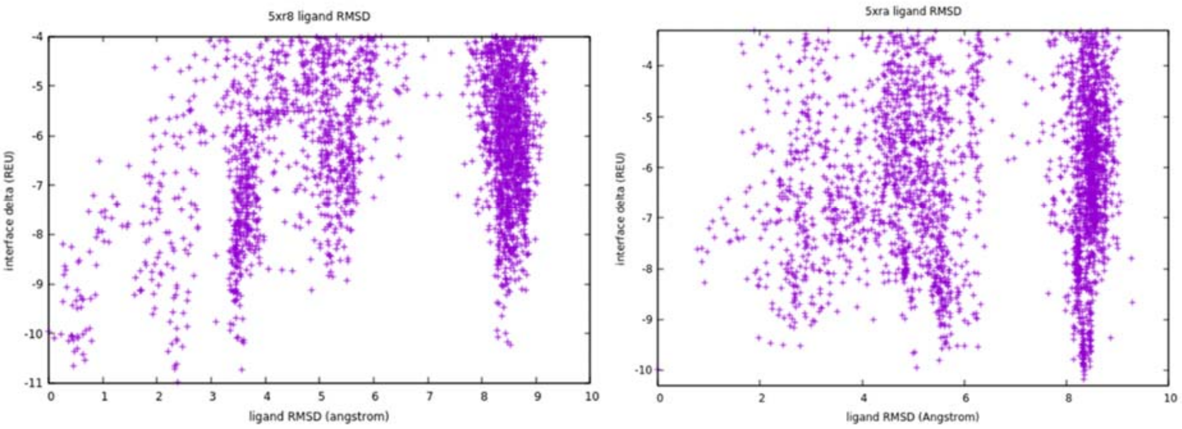

Figure S1: ligand RMSD versus Score (interface delta)

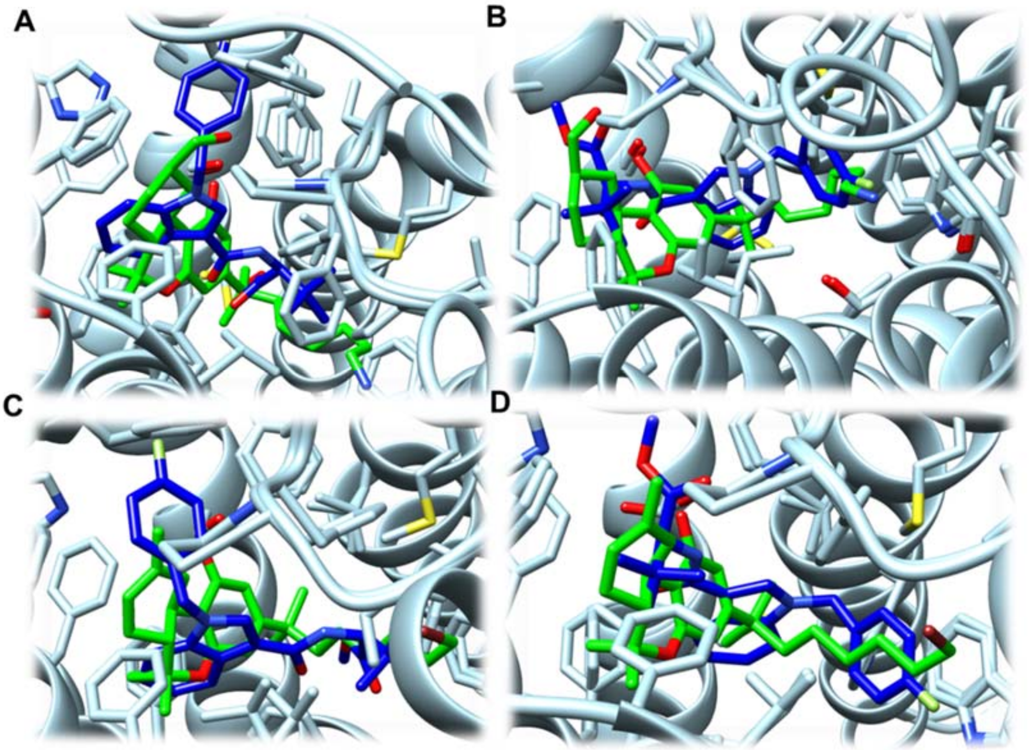

Figure S2: Poses obtained from docking. A and C show a pose, where the *N*-valine methyl ester aligns with the alkyl side chain of the THC-derivative, whereas B and D show poses, in which the *p*-fluorobenzyl residue aligns with the alkyl side chain of the THC-derivative. A and B are derived from 5xr8 and C and D from 5xra. Green = AM841/AM11542 (**45**), blue = MDMB-FUBINACA (**12**)

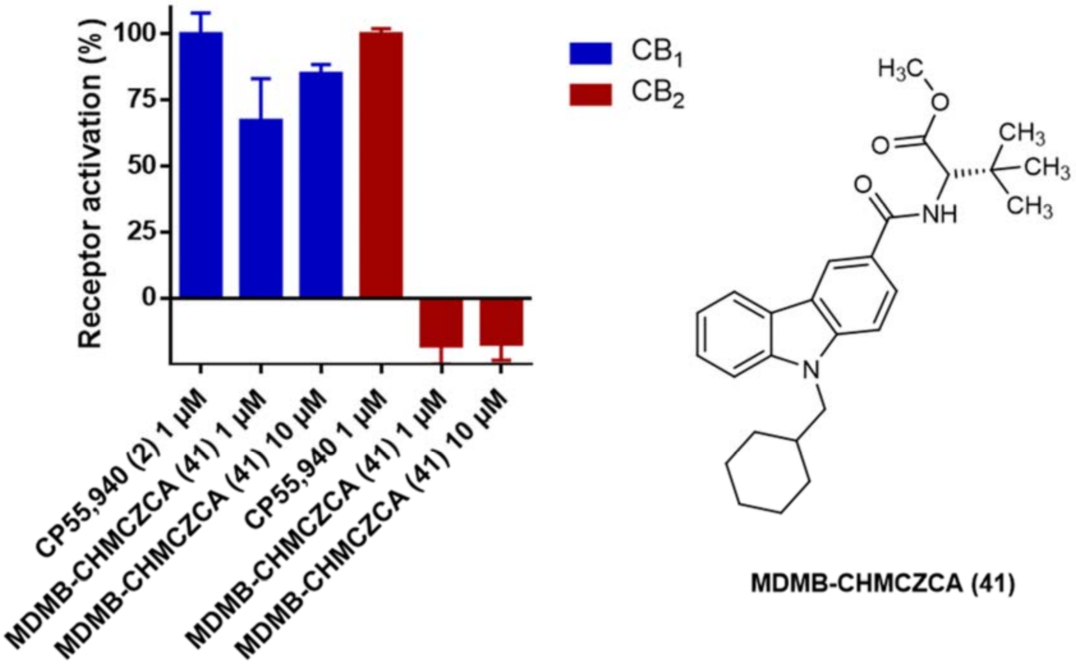

Figure S3: Cannabinoid receptor activation by MDMB-CHMCZCA (**41**) determined in cAMP accumulation assays

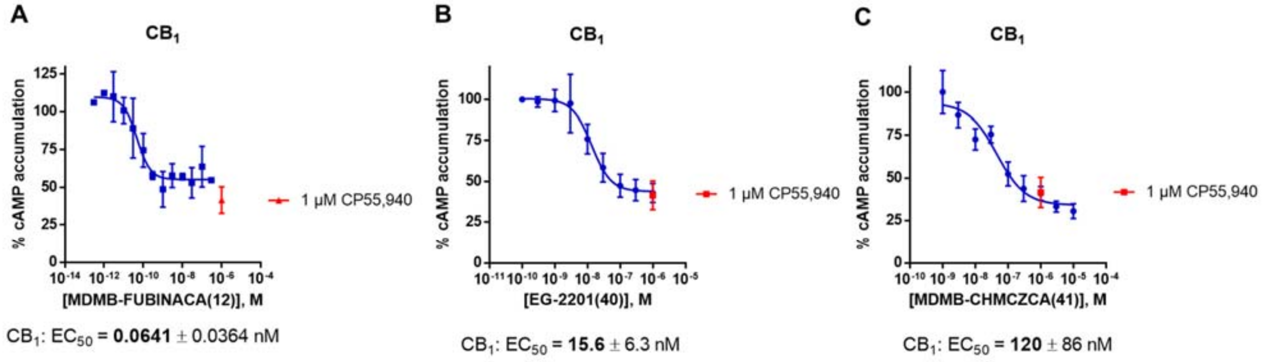

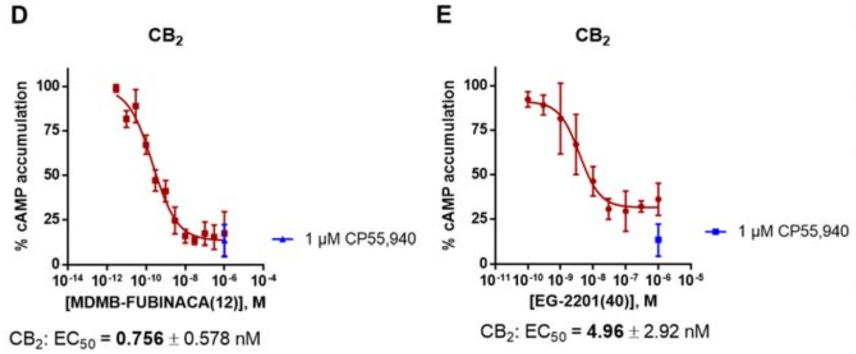

F

	Efficacy compared to max. response of CP55,940 (1 μM)
CB_1	
MDMB-FUBINACA (**12**)	82%
EG-2201 (**40**)	94%
MDMB-CHMCZCA (**41**)	116%
CB_2	
MDMB-FUBINACA (**12**)	100%
EG-2201 (**40**)	77%

Figure S4: Receptor activation of the cannabinoid receptor CB_1 (**A**-**C**) and CB_2 (**D**-**E**) by MDMB-FUBINACA (**12**, **A**, **D**), EG-2201 (**40**, **B**, **D**) and MDMD-CHMCZCA (**41**, **C**). **F.** Efficacy of tested compounds compared to max. response of CP55,940 (**2**)

References

(1) Meiler J, Baker D (2006) ROSETTALIGAND: protein-small molecule docking with full side-chain flexibility. Proteins 65: 538–548

(2) Lemmon G, Meiler J (2012) Rosetta Ligand docking with flexible XML protocols. Methods Mol Biol 819: 143–155

(3) McLachlan AD (1982) Rapid comparison of protein structures. Acta Crystallographica Section A 38: 871–873
